# Role of Contrast-Enhanced Ultrasonography in Hepatocellular Carcinoma by Using LI-RADS and Ancillary Features: A Single Tertiary Centre Experience

**DOI:** 10.3390/diagnostics11122232

**Published:** 2021-11-29

**Authors:** Adriana Ciocalteu, Sevastita Iordache, Sergiu Marian Cazacu, Cristiana Marinela Urhut, Sarmis Marian Sandulescu, Ana-Maria Ciurea, Adrian Saftoiu, Larisa Daniela Sandulescu

**Affiliations:** 1 Department of Gastroenterology, Research Center of Gastroenterology and Hepatology, University of Medicine and Pharmacy of Craiova, 200349 Craiova, Romania; adriana_ciocalteu@yahoo.com (A.C.); sevastita@gmail.com (S.I.); cazacu2sergiu@yahoo.com (S.M.C.); adriansaftoiu@aim.com (A.S.); larisasandulescu@yahoo.com (L.D.S.); 2 Department of Gastroenterology, Emergency County Hospital of Craiova, 200642 Craiova, Romania; cristiana.urhut@yahoo.com; 3 Department of Surgery, Emergency County Hospital of Craiova, University of Medicine and Pharmacy of Craiova, 200349 Craiova, Romania; ssarmis@yahoo.com; 4 Department of Oncology, Emergency County Hospital of Craiova, University of Medicine and Pharmacy of Craiova, 200349 Craiova, Romania

**Keywords:** contrast-enhanced ultrasonography, hepatocellular carcinoma, ancillary features, liver imaging reporting and data system

## Abstract

Clinical utility of ancillary features (AFs) in contrast-enhanced ultrasound (CEUS) Liver Imaging Reporting and Data System (LI-RADS^®^) is yet to be established. In this study, we assessed the diagnostic yield of CEUS LI-RADS and AFs in hepatocellular carcinoma (HCC). We retrospectively included patients with risk factors for HCC and newly diagnosed focal liver lesions (FLL). All lesions have been categorized according to the CEUS LI-RADS v2017 by an experienced sonographer blinded to clinical data and to the final diagnosis. From a total of 143 patients with 191 FLL, AFs favoring HCC were observed in 19.8% cases as hypoechoic rim and in 16.7% cases as nodule-in nodule architecture. From the total of 141 HCC cases, 83.6% were correctly classified: 57.4%- LR-5 and 26.2%- LR-4. In 9.21% cases, CEUS indicated LR-M; 2.12% cases- LR-3. The LR-5 category was 96.2% predictive (PPV) of HCC. LR-5 had 60.4% sensitivity and 93.6% specificity. PPV for primitive malignancy (LR-4 + LR-5) was 95.7%, with 88% sensitivity, 89.3% specificity and 88.4% accuracy for HCC. LR-4 category had 94.8% PPV and 26.2% sensitivity. CEUS LR4 + LR5 had 81,8% sensitivity for HCCs over 2 cm and 78.57% sensitivity for smaller HCCs. CEUS LR-5 remains an excellent diagnostic tool for HCC, despite the size of the lesion. The use of AFs might improve the overarching goal of LR-5 + LR-4 diagnosis of high specificity for HCC and exclusion of non-HCC malignancy.

## 1. Introduction

Liver tumors are a heterogeneous pathology with multiple variables, which are not uniform globally, making it difficult to establish a system of accurate diagnosis and prognosis.

Hepatocellular carcinoma (HCC) is among the first malignancies in terms of grim prognosis, being one of the neoplasms with a growing incidence [1]. HCC develops almost exclusively in the context of a chronic liver pathology and in about 90% of cases the patient has liver cirrhosis, the occurrence of which is determined by various etiologies. Chronic infections with hepatitis B and C viruses are recognized worldwide as the main factors involved in hepatocarcinogenesis, with the risk of developing HCC in infected patients being up to 30 times higher [2]. Accurate differential diagnostic is mandatory with other malignant lesions such as intrahepatic cholangiocellular carcinoma (ICC), which is the second most common primary malignant liver tumor that usually arises in healthy liver parenchyma, with different treatment approaches and prognosis [3].

Therefore, the optimization of minimally invasive, imaging and laboratory methods, which provide better tumor characterization, improve early diagnosis and prognosis of patients, is necessary. However, there is currently no single method to meet these conditions, despite multiple studies suggesting various combinations of serum biomarkers and imaging tests.

Contrast-enhanced ultrasound (CEUS), as a non-invasive imaging technique of describing focal liver lesions (FLL) according to features of their microvascularization, has proven to be an accurate diagnostic technique lately, comparable to contrast-enhanced magnetic resonance imaging (MRI), but faster and with a better safety profile [4]. The contrast agent approved for use at European level generates a high accuracy map of intratumoral vascularization and can help to assess even the degree of tumor differentiation [5].

CEUS has dramatically increased the capability of ultrasonography for the detection of FLL and has the potential to be incorporated into the diagnostic algorithm for malignant FLL [6].

Unfortunately, controversies such as the differential diagnosis of HCC and ICC with CEUS still exist [7]. Another limitation of the method is that it does not allow staging or detection of extrahepatic synchronous lesions as computed tomography (CT) and MRI do.

To date, the Liver Imaging Reporting and Data System (LI-RADS^®^) represents the most comprehensive system for imaging diagnosis, providing guidance on all imaging-related aspects of HCC, from technique for acquisition, reporting, assessment of treatment response and management [8]. Recently, LI-RADS diagnostic algorithms have been developed for CEUS and there are only a limited number of studies evaluating the diagnostic performance of CEUS LI-RADS [9].

LI-RADS guidelines define ancillary features (AFs) as imaging features that modify the likelihood that an observation is HCC and they were divided into those favoring malignancy in general, those favoring HCC in particular, and those favoring benignity [10]. Unlike in contrast-enhanced CT and contrast-enhanced-MRI, the use of AFs in CEUS is being evaluated, but studies are still limited. Their clinical utility is also yet to be evaluated.

The aim of this study was to assess the diagnostic yield of this imaging method recently introduced by using the newest LI-RADS algorithm and ancillary features, originally developed for CT and MRI, in the characterization and diagnosis of FLL and particularly of HCC. To the best of our knowledge, this is the first blind study of CEUS application in assessing FLL by using the LI-RADS algorithm and AFs.

## 2. Materials and Methods

We retrospectively included patients with risk factors for hepatocellular carcinoma with newly diagnosed focal liver lesions, hospitalized between January 2016 and December 2019 in the Gastroenterology Department of Emergency County Hospital of Craiova, Romania. Our study was approved by the Ethical Committee of the University of Medicine and Pharmacy of Craiova. We were granted permission to review medical records on-site, on a dedicated computer, as per the standard protocol for retrospective studies.

All the patients included in this study performed CEUS and the clinical data, imaging and laboratory investigations were available for re-evaluation. The risk factors we took into consideration according to current HCC guidelines [11] were liver cirrhosis of any etiology and non-cirrhotic HBV patients.

We limited the inclusion criteria to the presence of risk factors, newly discovered focal liver lesions, presence of clinical database and standard complete CEUS performance (arterial phase, portal venous phase and late phase), availability of an accepted diagnostic reference standard—either CT/MRI or histology where indicated, the opportunity of at least one-year follow-up.

The exclusion criteria were: previous treatment (either interventional or systemic therapy), lack of clinical database, contraindication of CEUS, incomplete CEUS performance or patients for whom the minimum registration criteria for CEUS interpretation, according to CEUS LI-RADS^®^ Technical Recommendations (American College of Radiology, CEUS LI-RADS v2017 Core), were not met: record continuous cine loop from bubble arrival through peak APHE, then record static images at 60 s and with every intermittent (every ~30 s).

CEUS was performed by using Hitachi VISION Preirus System (Hitachi Medical Corporation, Tokyo, Japan) between 2016 and 2017, then Hitachi-Aloka Preirus Arietta 70 (Tokyo, Japan) between 2017 and 2019, respectively. Contrast-enhanced imaging bearing low-mechanical-index cadence contrast pulse sequencing technology using as an ultrasound contrast agent SonoVue (Bracco, Geneva, Switzerland) was employed.

Diagnosis was established according to current guidelines either through histological analysis or based on CT/MRI scan.

Standard and contrast-enhanced ultrasound recordings were assessed by an EFSUMB level 3 sonographer, with more than 10 years experience in CEUS (>6000–10,000 ultrasound examinations) and who was blinded to clinical data and to the final diagnosis.

### 2.1. CEUS Interpretation

The aspects of the liver lesions from the patients included in our study were appreciated as follows: intensity (hypo-, iso- of hyperintensity), the pattern of arterial phase enhancement (diffuse, rim, peripheral globular) and characteristics of the late phase: the presence of washout, degree of washout (marked, mild), time of washout onset.

All lesions have been categorized according to the CEUS LI-RADS described by The American College of Radiology scheme (Contrast-Enhanced Ultrasound Liver Imaging Reporting and Data System [LI-RADS^®^]) [12]. The interpretation was established based on the description of arterial, portal and venous phases.

The AFs were taken into account. CEUS AFs in favor of benignity were size reduction or stability >2 years of the tumor. The malignancy aspects were: definite growth, nodulein nodule architecture and mosaic architecture, which are patterns that favor HCC in particular. The evidence of one or more AFs in favor of benignity downgraded by 1 category the LI-RADS score, while one or more AFs of malignancy upgraded by 1 category up to CEUS LR-4.

Lesions suggesting a cyst/hemangioma or hepatic fat deposition/sparing were classified as LR-1. Distinct isoenhancing solid nodule <10 mm standed for LR-2 group. LR-3 category included nodules with iso- or hypoenhancement in the arterial phase without late washout, regardless of their size or, in the instance of the occurrence of mild and late washout, only if <20 mm in size. LR-4 category also included lesions ≥10 mm with arterial phase hyperenhancement (excluded rim and globular peripheral), but without any washout. LR-5 category comprised nodules ≥10 mm with arterial phase hyperenhancement (either global or in part) followed by washout appearance that was mild in degree and late in onset. If the lesion could not be categorized due to image degradation or omission, it was classified as LR-NC.

### 2.2. Histopathological Assessment

Histopathological assessment was required in patients with unclear imaging diagnosis or with inconclusive findings at CEUS, CT and MRI imaging. It was performed either on a tissue sample of the lesion obtained by surgical resection or percutaneous US-guided biopsy whenever it was possible. The biopsy sample was obtained by using an 18-gauge core needle with a 16 mm or 22 mm throw (Bard-Magnum Biopsy Instrument MN1816; Bard Medical, Covington, GA, USA).

### 2.3. Statistical Analysis

The IBM program- Statistical Analysis Software Package (SPSS) version 20 was used for data processing and descriptive analysis. The program was also used to calculate the diagnostic performance of CEUS rendered by the following parameters: sensitivity, specificity, positive predictive value (PPV), negative predictive value (NPV), likelihood ratio and accuracy.

## 3. Results

**Figure 1:**
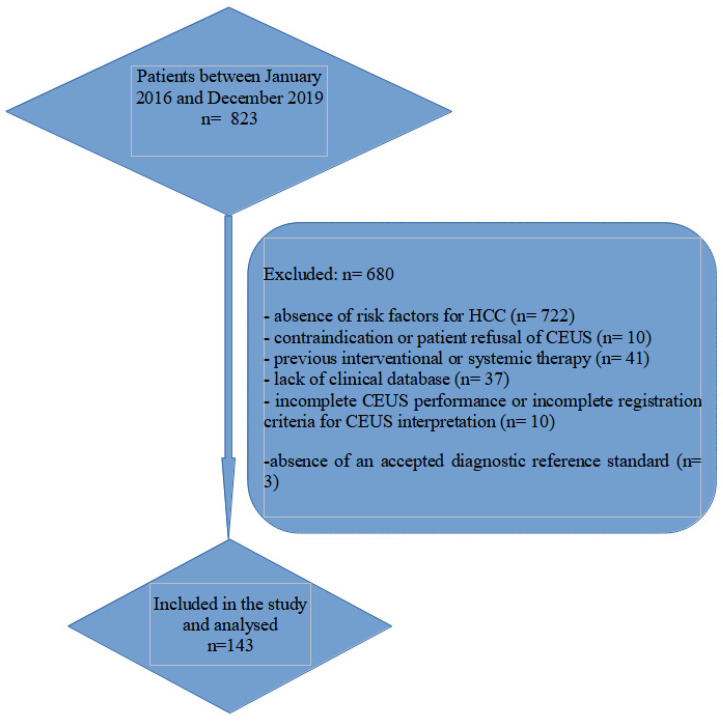
Flow chart of the patient enrollment.

This retrospective single-centre study included 143 patients with a total of 191 liver nodules examined from a total of 823 consecutive patients with FLL detected by standard abdominal ultrasound. The flow chart of the patients’ enrollment is shown in Figure 1. The distribution of patients by gender, age and etiology of liver disease is shown in Table 1. The majority of lesions were found in males (67.8%) and the average age at diagnosis was 65 years. The most common risk factor was liver cirrhosis which represented 90% of the identified etiologies. Hepatitis B virus (HBV) and hepatitis C virus (HCV) were detected in 67% of patients. In our study, most of the patients had one focal liver lesion. Forty-five nodules (23.5%) had the maximum diameter under 2 cm, 96 lesions (50.2%) were between 2 and 5 cm, while 50 of them (26.1%) were greater than 5 cm in size.

**Figure 2:**
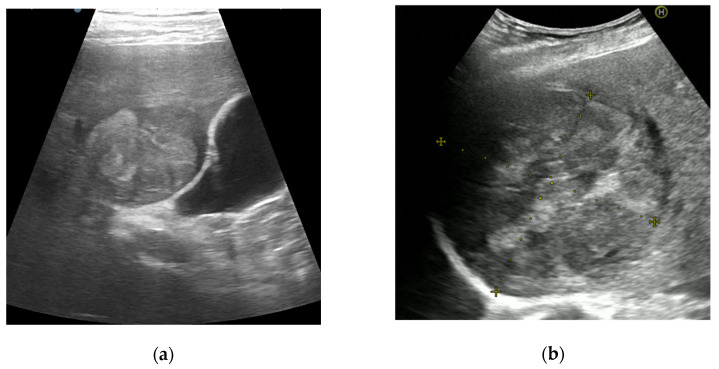
Illustrations of CEUS ancillary features in favor of HCC: nodule-in-nodule architecture (**a**) and mosaic architecture (**b**).

The presence of AFs favoring HCC was observed in 38 patients (19.8%) with a hypoechoic rim and in 32 patients (16.7%) with nodule-in-nodule architecture, respectively (Figure 2).

From a total of 191 lesions, the malignancy rate was 83%, representing 159 lesions. The distribution of diagnosis was as follows: 73% HCC (141 patients), 2% intrahepatic cholangiocarcinoma (ICC, 4 patients), 1% mixed tumor HCC/ICC (3 patients), 5% metastases from other primary sites and one malignant transformation of a hepatocellular adenoma (0.5%).

The majority of HCCs (n = 127; 66.4%) were greater than 2 cm in size, while only 14 HCCs (7.3%) were smaller than 2 cm from all nodules (90.07% vs. 9.9% from a total of 141 HCCs).

Furthermore, 32 benign tumors were diagnosed (16%): 19 regenerative liver nodules (9.9%), one focal nodular hyperplasia—FNH (0.5%), 6 liver hemangioma (3.1%) and 6 complex cysts (3.1%), respectively.

### 3.1. CEUS

Imaging features of all liver lesions on ultrasound are listed in Table 2.

**Figure 3:**
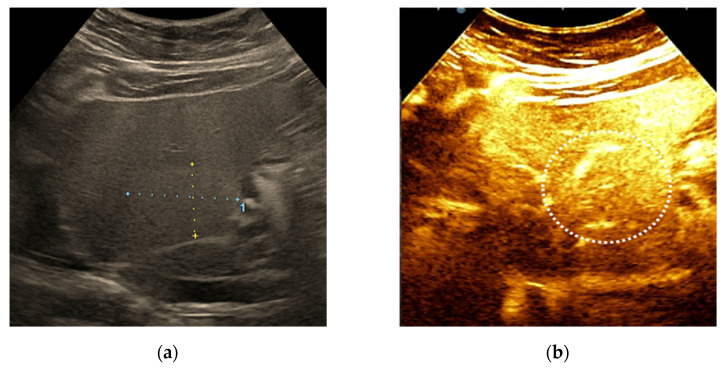

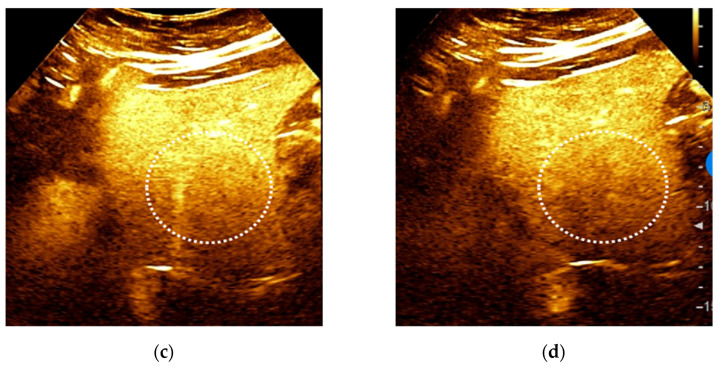
LIRADS 3 nodule in a chronic hepatitis B patient. An isoechoic liver nodule discovered on B-mode ultrasound (**a**) showed no APHE (**b**) and no washout of any type (**c**,**d**). Final histological diagnosis was HCC.

From the total of 141 *HCC* cases, 118 of them (83.6%) were correctly classified as HCC diagnosis, as follows: 81 tumors (57.4%)—definitely HCC (LR-5) and 37 tumors (26.2%)—probably HCC (LR-4). In 13 cases (9.21%), CEUS characteristics indicated malignant lesions, but not necessarily HCC (LR-M). Only 3 cases (2.12%) were classified as intermediate probability of malignancy (LR-3) and none of them was incorrectly diagnosed as benign (Figure 3).

As 3 of 4 *intrahepatic cholangiocarcinoma* were identified as LR-M, the histological analysis was required. Another ICC was inaccurately graded as LR-5. LR-M was properly established in all 3 cases of *mixed tumors HCC/ICCA*. All 10 *metastases* were also adequately graded as LR-M.

**Figure 4:**
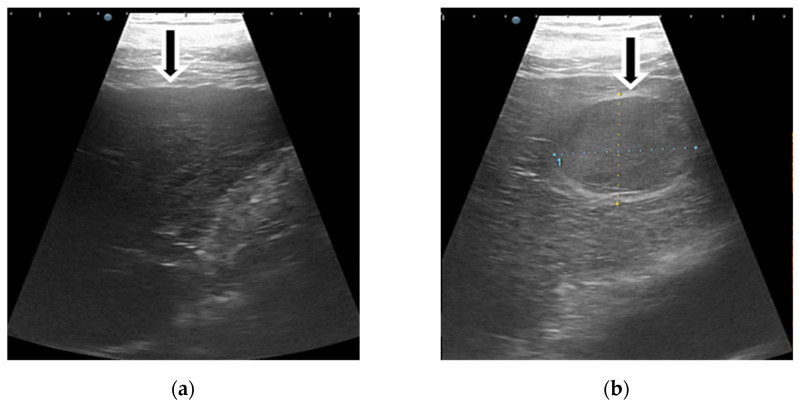

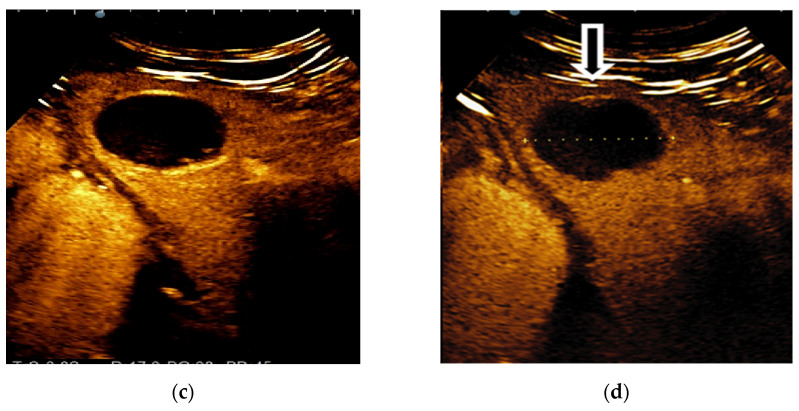
Illustration of LI-RADS-1 liver lesion in a patient with alcoholic liver cirrhosis. B-mode ultrasound using linear probe shows an inhomogeneous liver with nodular liver surface (**a**). In the right liver lobe, subcapsular, a well delimited, inhomogeneous hypoechoic liver lesion (**b**) that is nonenhancing on CEUS in all vascular phases (**c**,**d**). The final diagnosis was a complex cyst.

**Figure 5:**
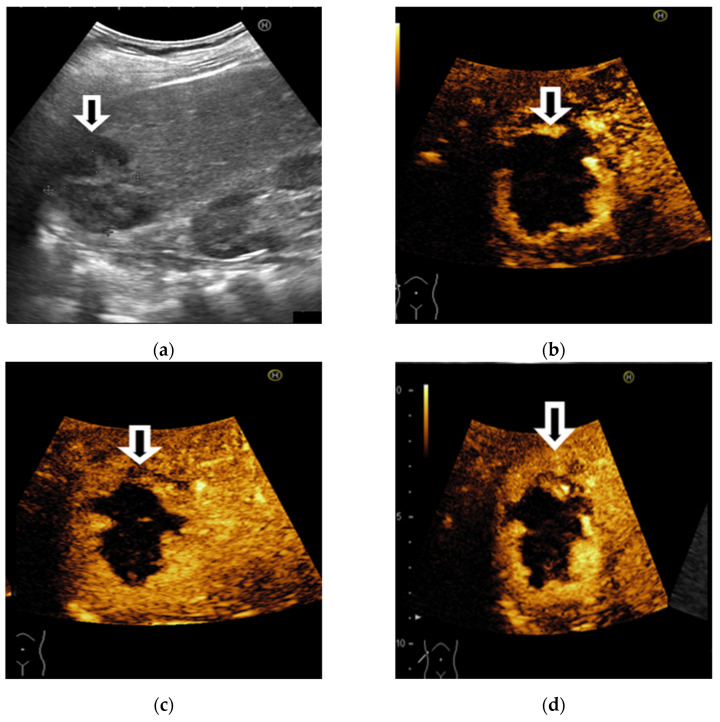

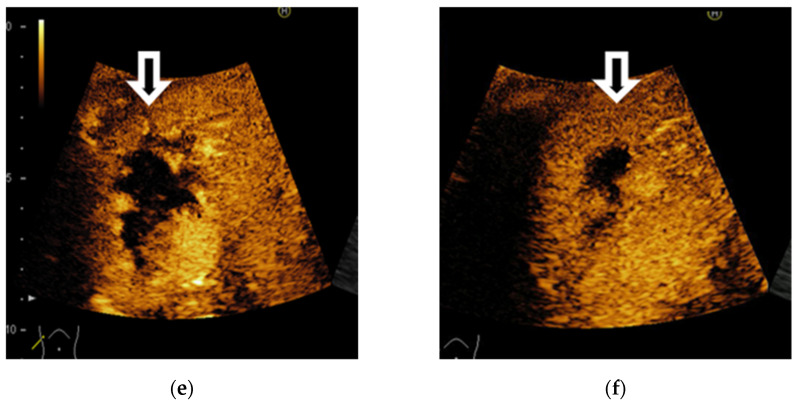
A case of focal liver lesions detected in a 68-year-old man with liver cirrhosis, characterised as LI-RADS-1 on CEUS. On B mode ultrasound is observed a hyperechoic inhomogeneous liver, nodular liver surface and a hypoechoic, inhomogeneous FLL in the right liver lobe (**a**). On CEUS, the liver lesion shows a typical early, peripheral, globular enhancement (**b**,**c**) and centripetal fill-in (**d**,**e**). In the late phase, incomplete enhancement is noticed (**f**).

CEUS provided correct grading as LR-1 for *FNH* and *complex cysts,* too (Figure 4*)*. From a total of 6 *hemangiomas*, 5 of them had typical, nodular, peripheral following by centripetal filling. Therefore, LR-1 grading was established (Figure 5). One hemangioma of 2 cm in size, with subcapsular location, had flash-filling aspect and false washout through rupture of gas bubbles inside the lesion immediately located below the ultrasound probe. That was the reason why it was misdiagnosed as probably HCC (LR-4). In this case, the correct diagnosis was established with CT and MRI.

From 191 lesions, 10 tumors (5.2%) were classified as not categorizable (LR-NC) because of the image degradation and, consequently, they were excluded from the *accuracy assessment*.

#### 3.1.1. CEUS Patterns According to LI-RADS Recommendations

The final diagnosis and the rates of different cellular types of nodules according to LI-RADS classes are listed in Table 3 and Table 4, respectively.

**Figure 6:**
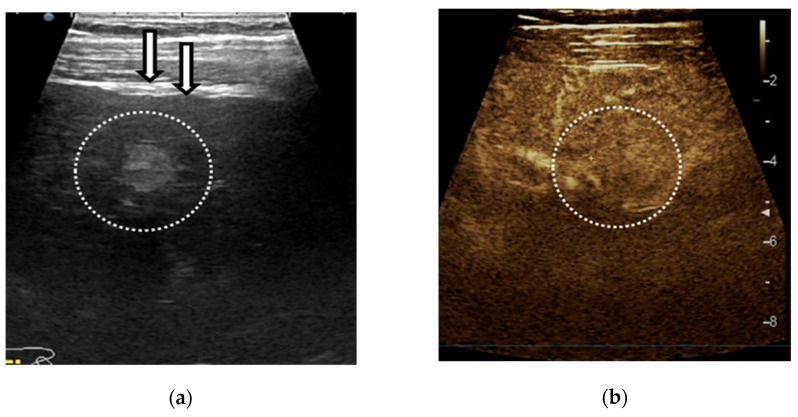

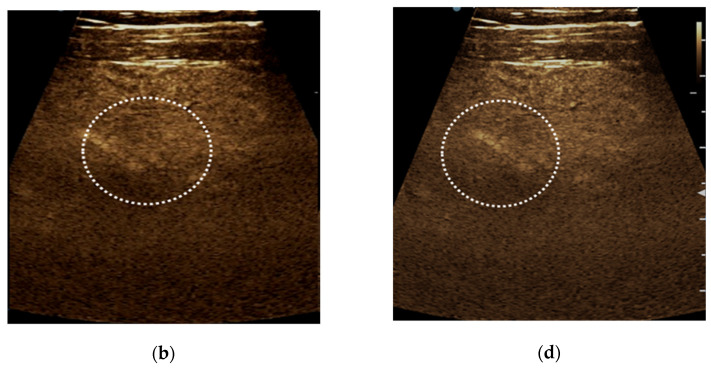
Example of LI-RADS-2 liver lesion. Regenerative nodule <10 mm depicted by linear probe exam in a patient with hepatitis C cirrhosis. The ultrasound exam shows a nodular liver surface and a subcapsular hyperechoic liver lesion (**a**). On contrast-enhanced ultrasound (CEUS) the nodule shows the isoenhancing aspect in the arterial (**b**), portal-venous (**c**) and late phase (**d**).

None of LR-1 (definitely benign) or LR-2 (probably benign) lesions (Figure 6) were finally diagnosed as malignant tumors. As a consequence of a flash-filling hemangioma, this was the only lesion with a false-positive diagnosis of malignancy from all LR-4, LR-5 or LR-M tumors.

**Figure 7:**
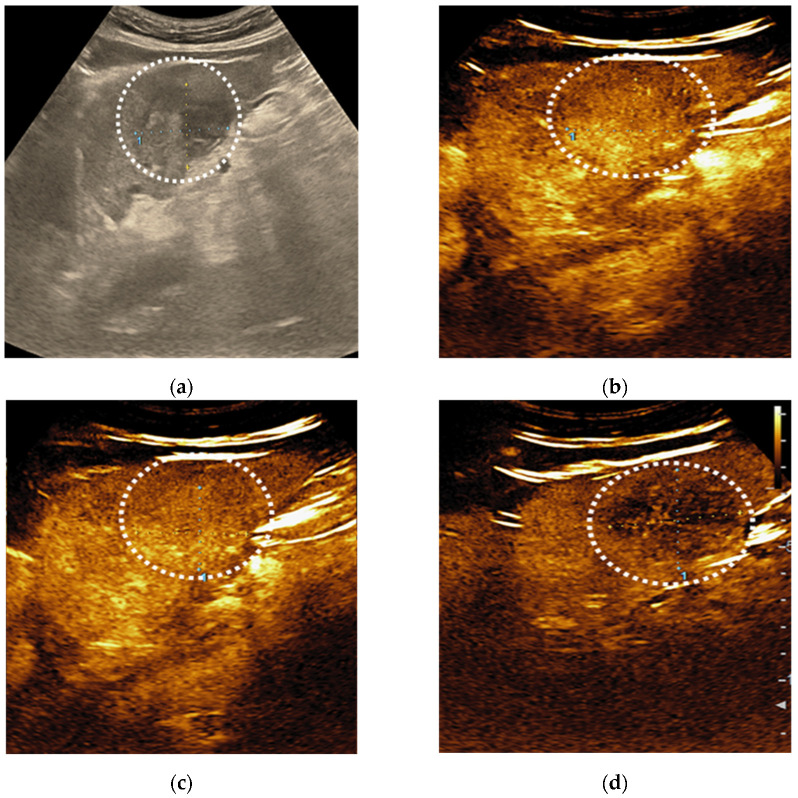
Hepatocellular carcinoma detected in a 54-year-old man with alcoholic cirrhosis, characterised as LI-RADS-5 on CEUS. On B mode ultrasound is observed an inhomogeneous lesion in the right liver lobe (**a**). On CEUS, the liver lesion shows an arterial phase enhancement (**b**) followed by washout appearance that was mild in degree and late in onset (**c**,**d**).

84 nodules (43.9%) were established as LR-5 and 81 of them (96.4%) were accurately diagnosed as HCC (Figure 7). The other 3 cases mistakenly graded were: one cholangiocarcinoma, one malignant transformed adenoma and one subcapsular hemangioma with flash-filling.

**Figure 8:**
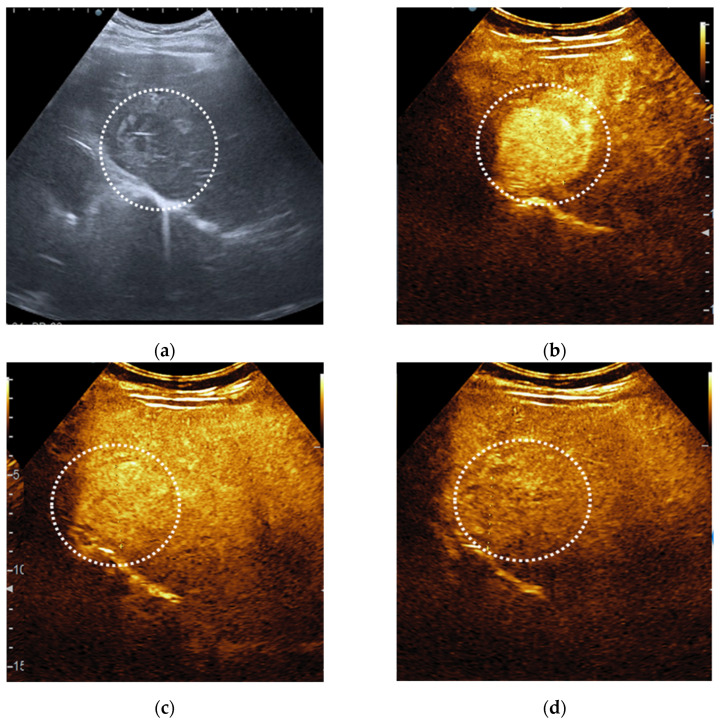
LI-RADS-4 liver lesion, probably HCC. B-mode ultrasound shows an inhomogeneous liver nodule (**a**). After intravenous administration of contrast agent, an arterial phase hyperenhancement (APHE) is observed (**b**). No washout is noticed in the portal (**c**) or in the late phase (**d**).

There were 37 lesions from 39 cases (94.8%) classified as probably HCC (LR-4) that were finally diagnosed as HCC (Figure 8).

**Figure 9:**
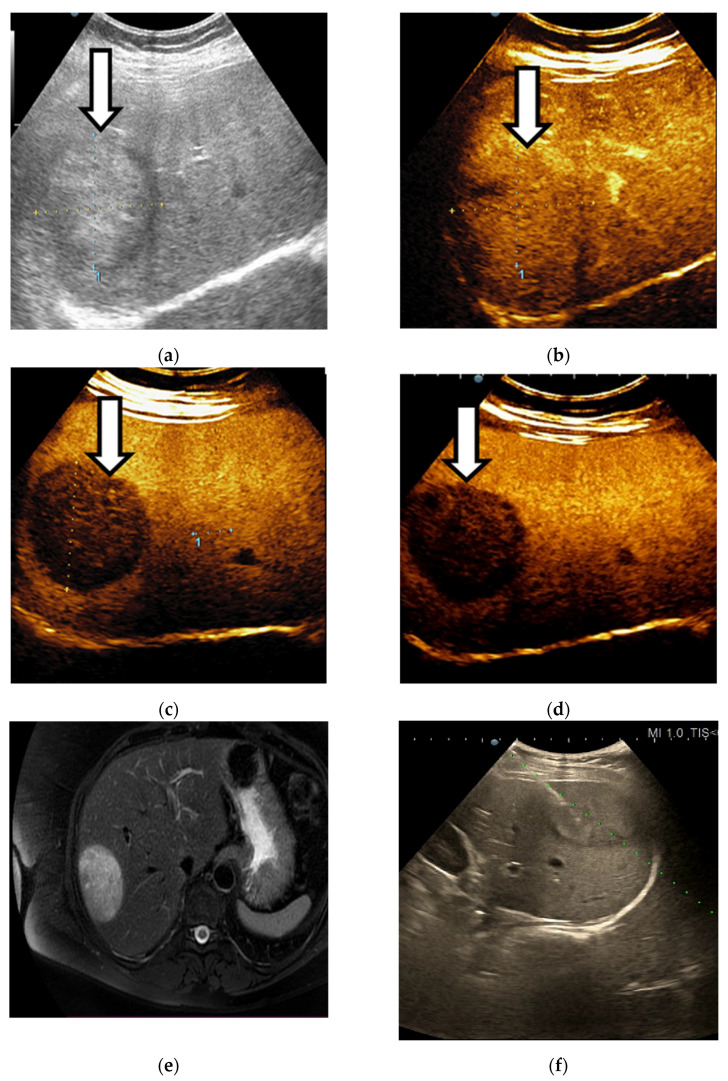

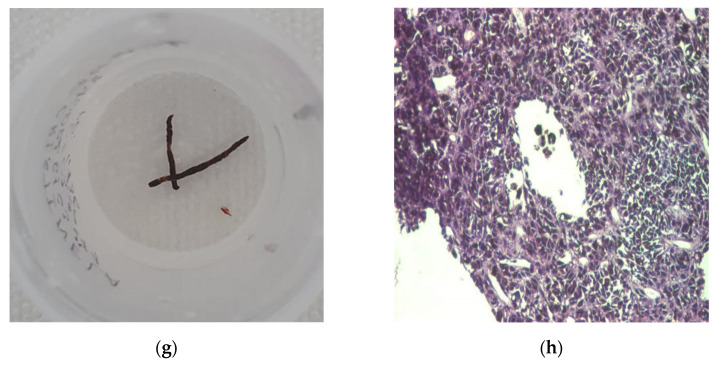
A case of liver metastases detected in a 64-year-old woman with chronic hepatitis B, LI-RADS-M aspect. The ultrasound exam shows a hyperechoic lesion with a halo in the right liver lobe (**a**). On CEUS, the liver lesion shows an arterial phase enhancement (**b**). In the portal phase an early washout was noticed (**c**), followed by marked washout in the late phase (**d**). The aspect of focal liver lesion on the CT scan (**e**). Echo-guided liver biopsy was performed (**f**). Two black liver fragments (**g**) with melanoma histological aspect were obtained (×40) (**h**).

There were 29 lesions classified by using CEUS as probably or definitely malignant but not HCC specific (LR-M); 13 of them (44.8%) were HCC, 3 of them (10.3%) were ICC, 3 (10.3%) mixed tumor HCC/ICC and 10 (34.4%) metastases (Figure 9).

#### 3.1.2. CEUS Accuracy

Sensitivity, specificity and accuracy for LI-RADS categories and overall diagnostic accuracy of LI-RADS patterns for HCC are reported in Table 5, Table 6 and Table 7, respectively. To calculate the accuracy for HCC, 10 nodules that were classified as not categorizable (LR-NC) were excluded, while LR-M nodules were taken into consideration as a false-negative.

Individually taken, the LR-5 category was 96.2% (95% CI: 89.4–98.7%) predictive (PPV) of HCC, with one case of misdiagnosis for cholangiocarcinoma. CEUS LR-5 sensitivity for HCC was 60.4% and specificity was 93.6%.

PPV for primitive malignancy (LR-4 + LR-5) was 95.7% (95 CI%: 90.7–98%), 88% sensitivity, 89.3% specificity and 88.4% accuracy for HCC (95CI%: 82.8–92.6%).

Regarding the LR-4 category, PPV was 94.8%, with only 26.2% sensitivity, whereas for LR-3 the PPV was 41.02%, with only 2% sensitivity.

CEUS LI-RADS L4 + L5 had 81.8% sensitivity for HCCs over 2 cm (n = 127), and 78.57% sensitivity for small HCCs less than 2 cm (n = 14).

### 3.2. Histopathologic Diagnosis

Histological diagnosis was available in 48 (25%) lesions. Thus, of the 36 cases of HCC, CEUS correctly classified 27 cases (81.8%) as probably (LR-4) or definitively HCC (LR-5). All 3 cases of ICC, 3 cases of mixed tumor (HCC/ICC) and 4 biopsied metastases were correctly defined by CEUS as lesions probably or definitely malignant but not specific HCC (LR-M).

## 4. Discussion

To date, CEUS is considered to be a second-line imaging technique in the characterization of FLL, having the advantage of a low cost. Beyond contrast-enhanced MRI as the method of choice for characterizing FLL, there are studies concluding that there are no statistically significant differences between CEUS and MRI or CT [9,13,14]. However, the differential diagnosis between HCC, cholangiocarcinoma and liver metastases may be limited by similarities in the appearance of CEUS. As there is moderate evidence, the challenges of characterizing multiple nodules and comparing with CT or MRI examinations, EASL provided a weak recommendation in favor of using CEUS for the diagnosis or monitoring of HCC [11].

Studies focused on AFs in MRI and there is little evidence on their use in CEUS or on the differences between the use of different contrast agents. To our knowledge, this is the first blind evaluation of CEUS performance by highly experienced in abdominal ultrasound and CEUS by using the latest LI-RADS algorithms and AFs.

This study highlights that the CEUS LR-5 pattern by using this new LI-RADS algorithm remains an excellent diagnostic tool for HCC. Sensitivity and specificity for diagnosing HCC of CEUS LR-5 (60.4% and 93.6%, respectively) were quite similar to the estimates of CT / MRI LR-5 (sensitivity 67% and specificity 93%) [9], and also PPV is very similar to the conclusions from a large multicenter study where AFs were not taken into consideration (PPV 96.2% vs. 98.5%) [15]. As for the LR-4 pattern, similar sensitivity was reported (26.2% vs. 21%), but we obtained a more significant PPV than in the above-mentioned study (94.8% vs. 86%). Thus, the rate of lesions classified as probably HCC (LR-4) by CEUS, that were finally diagnosed as HCC, was also similar with the rate of LR-4 observations on MRI that were visible on US and that were determined to be HCC nodules (94.8% vs. 96%) [16]. In our cohort, one CEUS false positive in LR-4 group was a hemangioma with flash-filling aspect and false washout due to the rupture of gas bubbles inside the lesion immediately located below the ultrasound probe, which was correctly diagnosed by CT and MRI.

Nevertheless, Schellhaas et al. suggested the combination of the categories LR-4 and LR-5 for the diagnosis of definite HCC to improve the CEUS LI-RADS v2017 algorithm [17]. Indeed, in our study, both sensitivity and accuracy of combined LR-4 and LR-5 patterns for the diagnosis of definite HCC considerably raised to 88.07% and 88.4%, unlike the specificity and accuracy for LR-5 of only 60.45% and 69%, respectively. In this situation, NPV also improved (73.4% vs. 46.6%), while similar high PPVs (95.7% vs. 96.2%) and quite similar specificity (89.3% vs. 93.6%) were maintained. On the other hand, in another recent study from a single tertiary centre, where the use of AFs was not mentioned, the combination of LR-4 and LR-5 had a significant lower specificity than LR-5 alone [18]. Therefore, the use of AFs might improve the overarching goal of CEUS LR-5 + LR-4 diagnosis of high specificity for HCC and exclusion of non-HCC malignancy.

Interestingly, higher sensitivity than estimated for the diagnosis of HCCs smaller than 2 cm was achieved for CEUS LR-4 and LR-5 (78.5%), which was similar to CEUS sensitivity for HCCs over 2 cm (78.9%) in another study [19]. Therefore, in our case, the CEUS LI-RADS algorithm had good accuracy for the diagnosis of HCC, despite the size of the lesion. The combination of LR-5 and LR-4 may need further discussion and adjustment in CEUS LI-RADS studies including AFs in order to decide its utility in HCC guidelines.

PPV for LR-3 category for nodules at intermediate risk of HCC was 41.02%, which was close to 50% considered as the middle risk and to Terzi et al.’s results (47%), but with a lower sensitivity of only 2% [15]. Anyway, there was a low rate of different cellular types of nodules according to LI-RADS classes included in the LR-3 category (3 CHC and 5 non-recognizable because of the image degradation).

It is still controversial whether CEUS can make a specific diagnosis of HCC because of the potential risk of misdiagnosis in the case of ICC which manifest global arterial phase hyperenhancement followed by washout at CEUS, leading to a misdiagnosis of HCC in approximately 50% of the cases [20,21,22]. Taking into consideration the risk of misdiagnosis of ICC for HCC as the main reason for removing CEUS from the HCC guidelines, in our approach, histological diagnosis proved that all ICCs were correctly defined by CEUS as lesions probably or definitely malignant but not specific HCC (LR-M), with one exception that it was inaccurately graded as LR-5. Also, all 10 metastases, including 4 biopsied metastases, were adequately graded by CEUS as LR-M. Our results are in agreement with a recent meta-analysis stating that CEUS did not increase the risk of misdiagnosing other malignancies as HCC when compared to CT/MRI [9]. Even if HCC and ICC can present either as a hyperenhanced lesion or as a hypoenhanced lesion during the arterial phase, a situation that poses problems of differential diagnosis with liver metastases [23,24,25,26], a peculiarity of liver metastases is the presence of an area of peripheral hyperenhancement in the arterial phase, which combined with rapid washout in the portal phase can lead to the correct diagnosis.

All LR-M from our cohort were confirmed as malignancies, therefore with better accuracy than that of the CT/MRI LR-M (93%) category reported in a previous meta-analysis [27]. HCCs represented 44.8% of the LR-M group, while the proportion of other malignancies was 55.1%, which was also higher than in other CEUS LI-RADS studies [28,29], but similar to CT/MRI LI-RADS (57–77%) [30]. This could be associated with geographic differences in the dominant etiologies of underlying liver disease, which is in accordance with studies from Western countries which had a lower proportion of HCCs and a higher proportion of non-HCC malignancies in the CEUS LR-M category than those from Asian countries [29]. Furthermore, in our cohort, 48.2% of non-HCC malignant nodules had suggestive early washout <60 s. In a recent meta-analysis, the majority of all CEUS LR-M lesions were malignancies (94%), with HCCs representing 54% and non-HCC malignancies representing 40%. The frequencies of individual CEUS LR-M imaging features varied; early washout showed the highest frequency for non-HCC malignancies [29].

To date, there are no subgroup analyses based on major and ancillary features of the CEUS LI-RADS because of insufficient data [9].

From all the benign tumors that were diagnosed correctly, only one lesion was a focal nodular hyperplasia, FNH (0.5%), in a female patient, 65 years old, with alcoholic liver cirrhosis. The appearance of the nodule was characteristic, filling with contrast medium from the center to the periphery, originating from a central arterial blood supply and we graded it as LR-2. FNH on liver cirrhosis is rarely reported. Despite the fact that this aspect could be a feature in favor of benignity, it is not recognized as a LI-RADS AF yet and it is considered of little value in daily practice, except for patients who are at risk for HCC due to hepatitis B without cirrhosis [10,31].

Therefore, the accuracy of blinded evaluation of CEUS by taking into consideration AFs was similar to other studies where prior to performing a liver CEUS study, the patient’s clinical history, laboratory data and any imaging findings were reviewed. Also, the sensitivity was not influenced by the size of the nodules.

The use of tumor size reduction as an AF in favour of benignity is questionable, as diameter reduction could happen secondary to the resorption of haemorrhage or the development of fibrosis [10]. Size change was not a limitation in our study as the only AFs noticed were hypoechoic rim (19.8%) and nodule in nodule architecture (16.7%). Anyway, updated LI-RADS guidelines for CEUS should take into consideration to optimize this criterion for use as an AF.

One limitation of this study regarding the assessment of AFs could be the fact that CEUS was perfomed by using only one contrast agent (SonoVue), while other agents could provide additional Kupffer phase images [10,28]. Future studies should assess AFs through comparing different contrast agents. Other limitations are the retrospective nature of the study, limitation to a single tertiary center that could prone the study to bias, possible subjective interpretation by the investigator who evaluated CEUS images and few lesions with histological diagnosis.

In conclusion, CEUS could be accepted by unanimous consent in international guidelines as a useful non-invasive tool with high accuracy to rapidly orientate liver tumor diagnosis, especially in high-risk patients, whenever it is available and performed by experienced sonographers. Given that CEUS LI-RADS and CEUS AFs are newly introduced concepts, more prospective multicenter cohort studies which assess the usefulness of this algorithm will bring important information for possible improvements in the future.

## Figures and Tables

**Table 1:** Patient characteristics.

Patient Characteristics (n = 143)	
Variable	n (%)
Patient’s gender, male	97 (67.8%)
Patient’s age, median (years) (±SD)	65.7 ± 8.17
Liver cirrhosis	129 (90.2%)
Etiology of liver disease, n (%)	
1. Alcohol	29 (20.2%)
2. Non-alcoholic steatohepatitis (NASH)	5 (3.49%)
3. HCV	55 (38.4)
4. HBV	41 (28.7)
5. Multiple etiology	6 (4.1%)
6. Other	4 (2.7%)
History of extrahepatic malignancy	8 (5.5%)
Number of lesions	
solitary lesion	111 (77.6%)
2–3 lesions	27 (18.8%)
>3 lesions	3 (2.09%)
diffuse tumor infiltration	2 (1.39%)

**Table 2:** Ultrasound features.

Ultrasound Features (n = 191)	
size of index lesion (n = 191)	(mean ± SD) 41.6 ± 27.1 mm
<2cm	45 (23.5%)
2–5 cm	96 (50.2%)
≥5 cm	50 (26.1%)
echo texture of index lesion (n = 191)	
hypoechoic	77 (40.3%)
isoechoic	20 (10.4%)
hyperechoic	94 (49.2%)
homogeneity of index lesion (n = 191)	66 (34.5%)
presence of hypoechoic rim	38 (19.8%)
noduleinnodule architecture	32 (16.7%)
mosaic architecture	34 (17.8%)
macroinvasion of liver veins/portal vein (B-mode, color mode)	26 (13.6%)

**Table 3:** The final diagnosis according to LI-RADS classes.

LI-RADS Classes (n = 191)		
The Final Diagnosis	n (%)	LI-RADS Classes
Malignant	159 (83.2%)	
HCC	141 (73.83%)	LR-5 = 81 (57.4%) LR-4 = 37 (26.2%) LR-3 = 3 (2.12%) LR-M = 13 (9.21%) LR-NC = 7 (4.96%)
ICC	4 (2.09%)	LR-M = 3 LR-5 = 1
mixed tumor (HCC/ICC)	3 (1.57%)	LR-M = 3
metastases	10 (5.23%)	LR-M = 10
malignant transformation of hepatocellular adenomas	1 (0.52%)	LR-5 = 1
Benign	32 (16.7%)	
regenerative/dysplastic nodule	19 (9.9%)	LR-1 = 3 LR-2 = 6 LR-3 = 5 LR-4 = 2 LR-NC = 3
FNH	1 (0.5%)	LR-2 = 1
hemangioma	6 (3.1%)	LR-1 = 5 LR-5 = 1
complex cyst	6 (3.1%)	LR-1 = 6

HCC—hepatocellular carcinoma; ICC—intrahepatic cholangiocellular carcinoma; FNH—focal nodular hyperplasia.

**Table 4:** The rates of different cellular types of nodules according to LI-RADS classes.

LI-RADS	Number of Lesions (n = 191)	Diagnosis
LR-1	14	Complicated cysts = 6 Hemangioma = 5 Regenerative Nodules = 3
LR-2	7	FNH = 1 Regenerative Nodules = 6
LR-3	8	HCC = 3 Regenerative Nodules = 5
LR-4	39	HCC = 37 Regenerative Nodules = 2
LR-5	84	HCC = 81 ICC = 1 Flash-filling hemangioma = 1 Adenoma with malignant transformation = 1
LR-M	29	HCC = 13 Mixed tumor = 3 ICC = 3 Metastases = 10
LR-NC	10	HCC = 7 Regenerative Nodules = 10

**Table 5:** LI-RADS-5 accuracy for HCC.

Statistic	Value	95% CI
Sensitivity	60.45%	51.64–68.78%
Specificity	93.62%	82.46–98.66%
Positive likelihood ratio	9.47	3.14–28.55
Negative likelihood ratio	0.42	0.34–0.53
Disease prevalence	73.00%	
PPV	96.24%	89.47–98.72%
NPV	46.68%	41.21–52.23%
Accuracy	69.40%	62.13–76.02%

PPV—Positive predictive value; NPV—Negative predictive value.

**Table 6:** LI-RADS-4 and 5 accuracy for HCC.

Statistic	Value	95% CI
Sensitivity	88.06%	81.33–93.02%
Specificity	89.36%	76.90–96.45%
Positive likelihood ratio	8.28	3.61–19.00
Negative likelihood ratio	0.13	0.08–0.21
Disease prevalence	73.00%	
PPV	95.72%	90.70–98.09%
NPV	73.46%	63.36–81.58%
Accuracy	88.41%	82.83–92.68%

**Table 7:** Diagnostic accuracy of LR patterns for HCC.

CEUS LI-RADS	Sensitivity (%)	PPV (%)
LR-3	2.10	41.02
LR-4	26.20	94.87
LR-5	60.45	96.24
LR 4 + 5	88.06	95.72

## Data Availability

The data presented in this study are available on request from the corresponding author and will be made available on the University’s repository as an anonymized dataset at a later date.
